# Supplementary figures and images for: Transepithelial transport of P-glycoprotein substrate by the Malpighian tubules of the desert locust
Source: PLoS One. 2019 Oct 8;14(10):e0223569. doi: 10.1371/journal.pone.0223569 (PMC6782089; doi:10.1371/journal.pone.0223569)

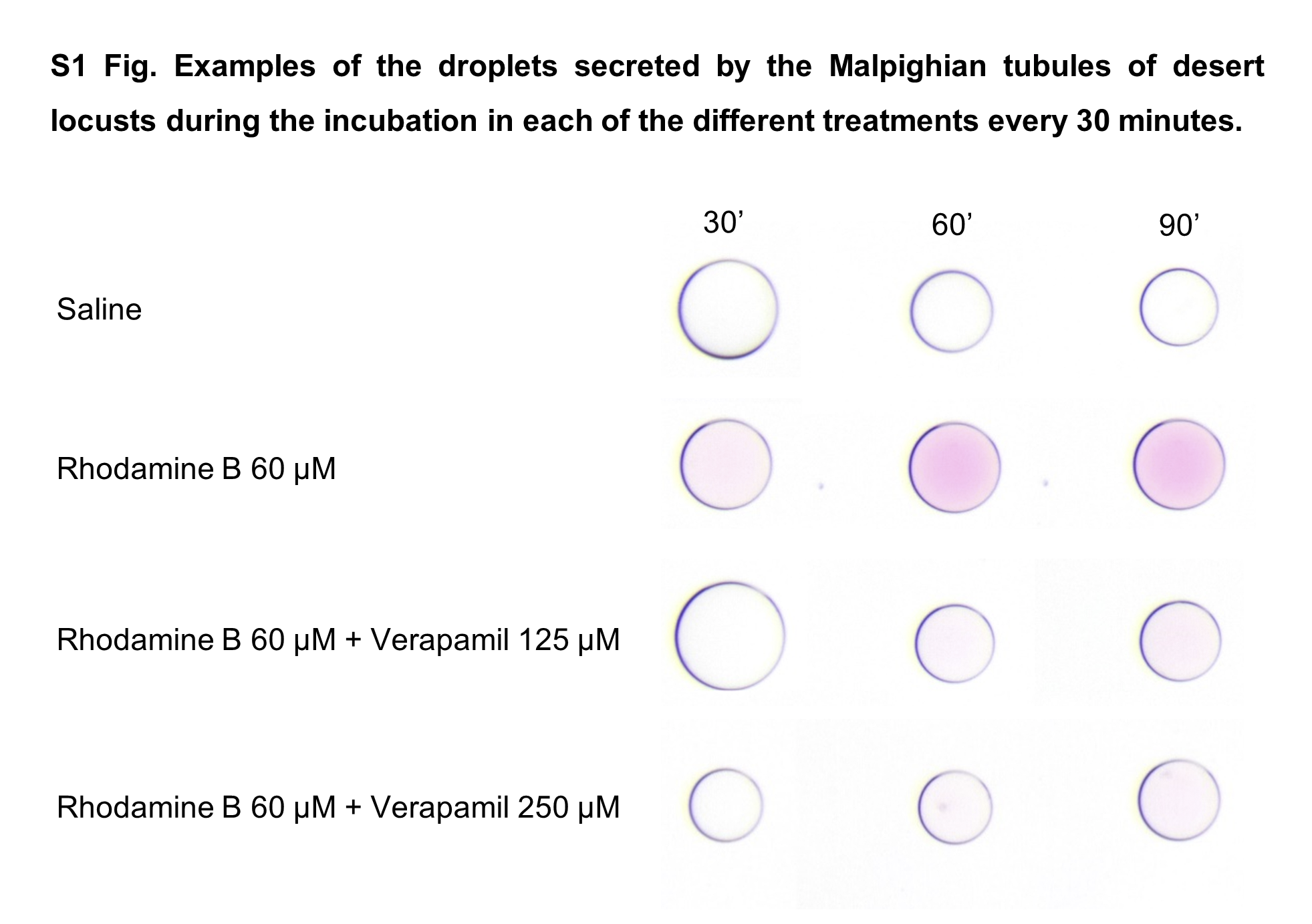

Supplement: S1 Fig — The size of each droplet depends upon the fluid secretion rate whilst the colour is determined by the net extrusion rate of rhodamine B. (TIF) [file pone.0223569.s001.tif]

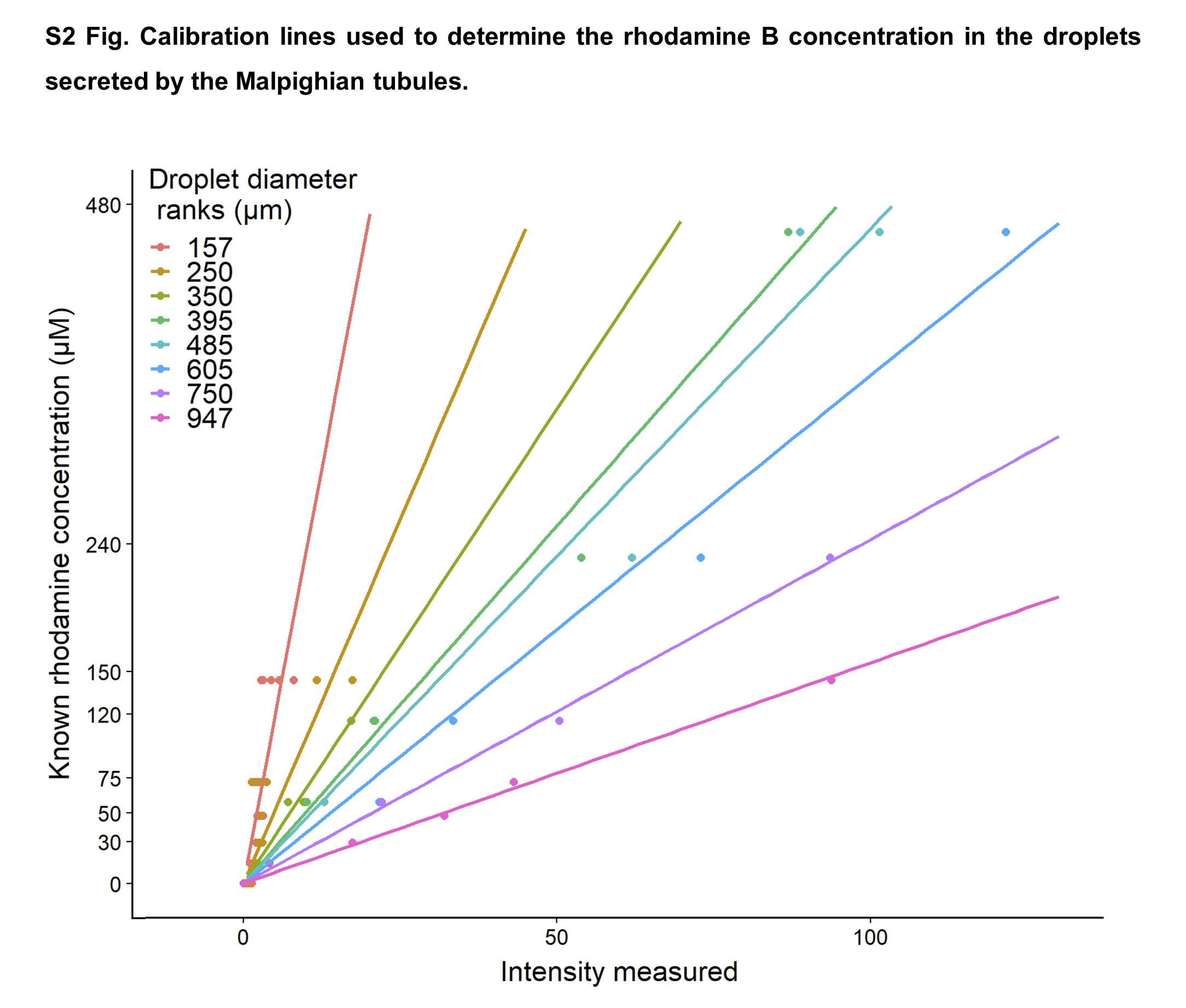

Supplement: S2 Fig — For each droplet diameter rank there is a linear relationship between the rhodamine concentration of the droplet and the colour intensity measured. The slope of the lines decreases as the diameter increases. We estimated the rhodamine B concentration of the droplets secreted by measuring the colour intensity and the diameter of each droplet. Each line represents the linear regression fit for each mean diameter rank. (TIF) [file pone.0223569.s002.tif]

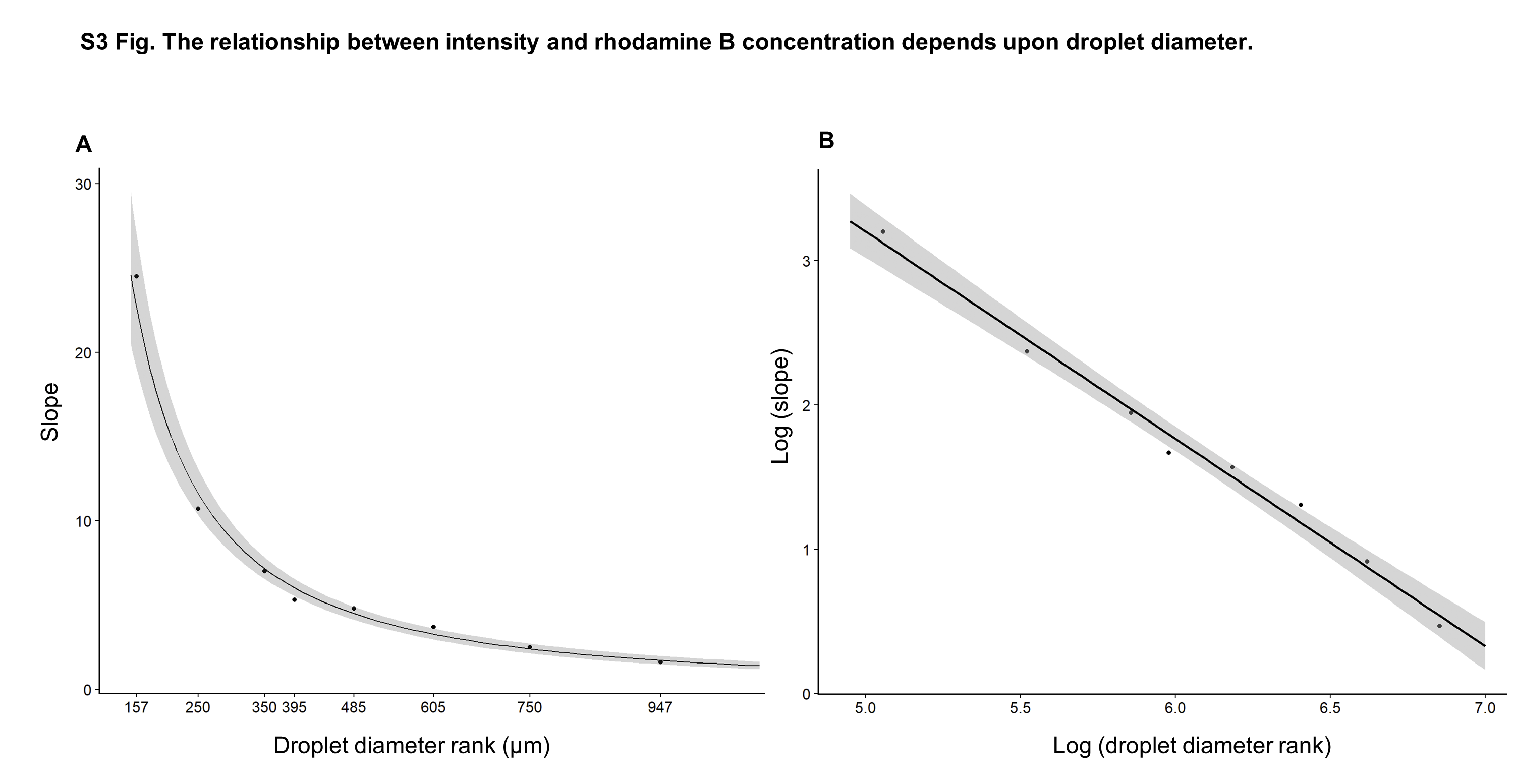

Supplement: S3 Fig — (A) The slope decreases as the diameter increases, following an exponential decay. (B) After log transformation the relationship becomes linear. Using this linear equation for each droplet diameter measured, we predicted the slope of the line equation that link the colour intensity to the rhodamine concentration. (TIF) [file pone.0223569.s003.tif]

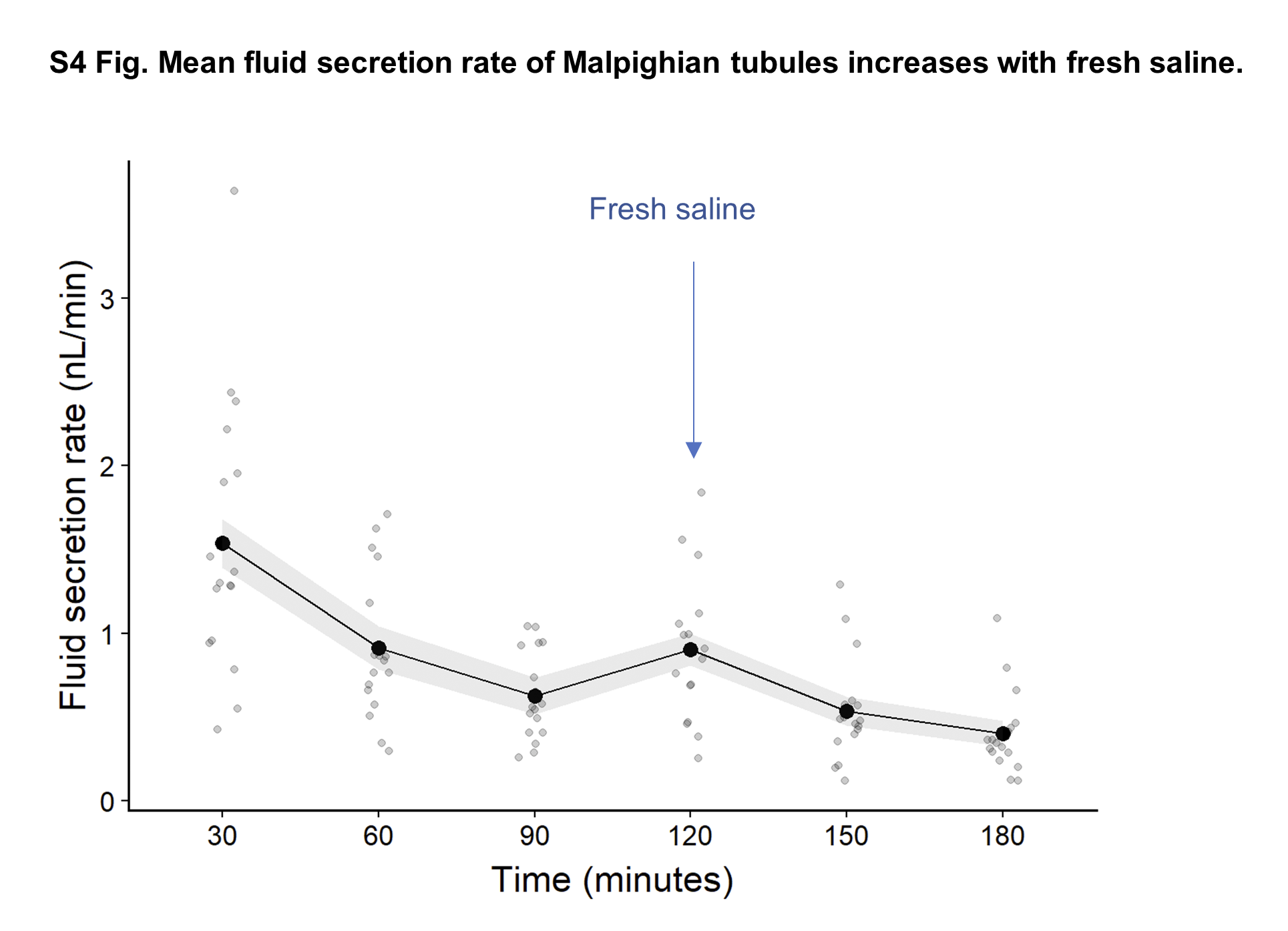

Supplement: S4 Fig — The tubules were incubated in saline but after 90 minutes the saline bath was removed and replaced with fresh saline. The arrow indicates the first measurement taken after the saline had been replaced. Grey points indicate the fluid secretion rate of individual tubules at a particular time point. (TIF) [file pone.0223569.s004.tif]

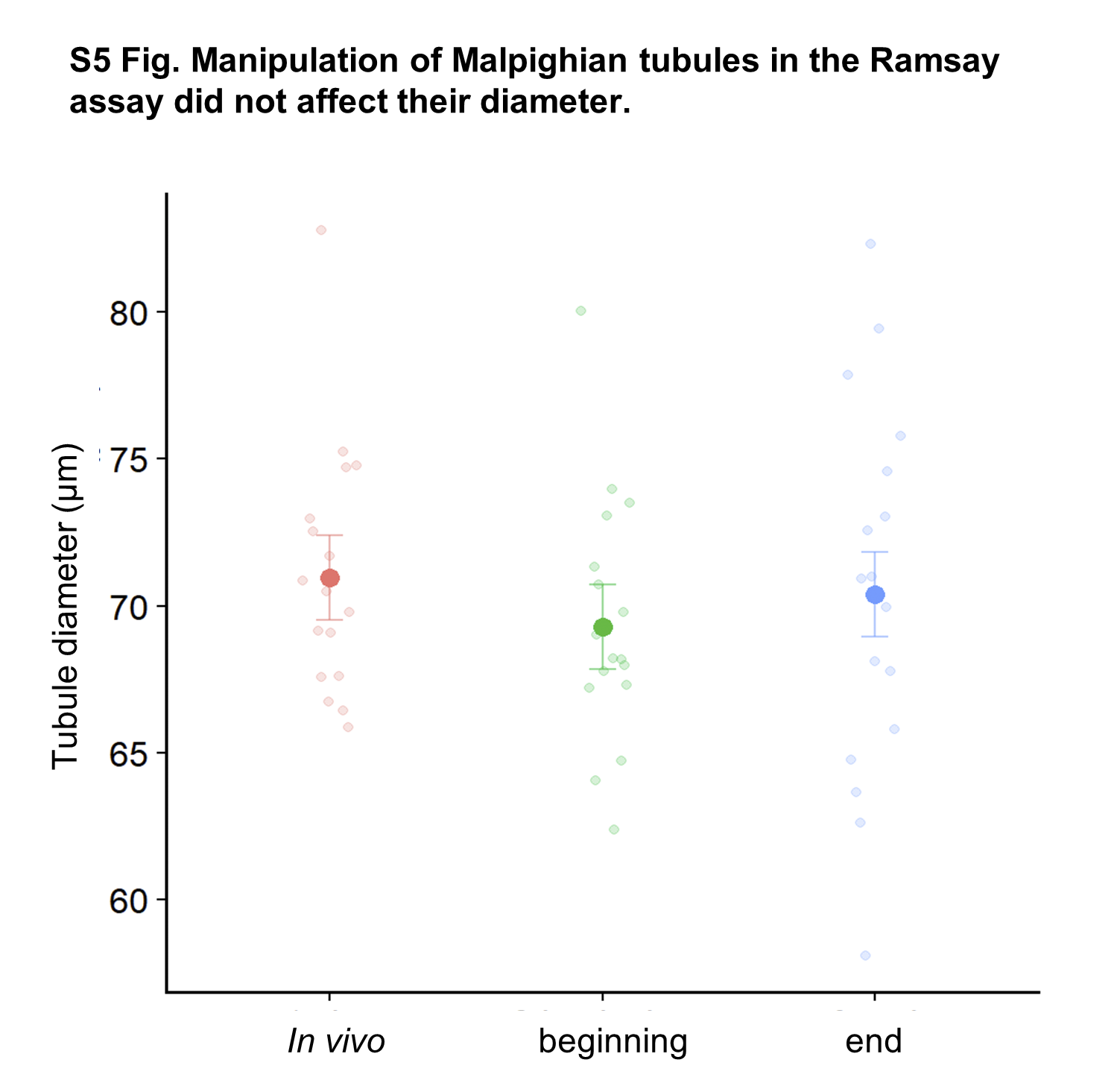

Supplement: S5 Fig — To exclude the possibility that manipulation during the assay affected tubule morphology, we measured the tubule’s diameter in vivo, at the beginning, and at the end of the assay. The diameter was unaffected by the manipulation. (TIF) [file pone.0223569.s005.tif]

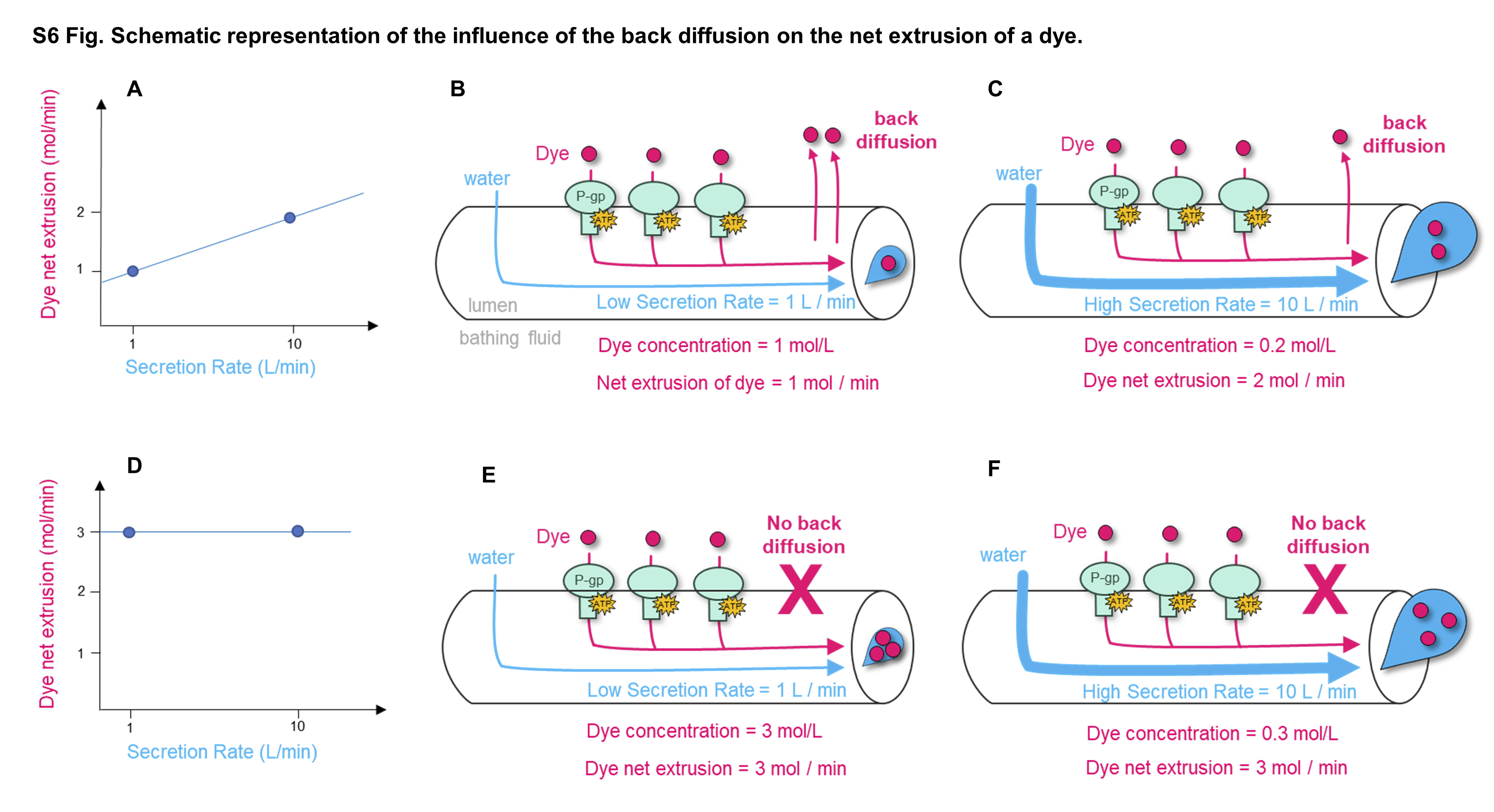

Supplement: S6 Fig — (A) The net extrusion of a dye positively correlates with the fluid secretion rate when the dye can back diffuse from the lumen to the bathing fluid. (B) Indeed, at low fluid secretion rate, the dye concentration in the lumen rises, increasing the back diffusion and reducing the net transport of the dye. (C) Instead, at higher fluid secretion rates, the dye concentration in the lumen is diluted and the back diffusion is reduced, increasing the net transport of the dye. (D) If no back diffusion occurs, there is no correlation between the net transport of the dye and the fluid secretion rate. (E,F) At any rate of fluid secretion, the net transport of the dye remains constant, independently of the dye concentration in the lumen. (TIF) [file pone.0223569.s006.tif]

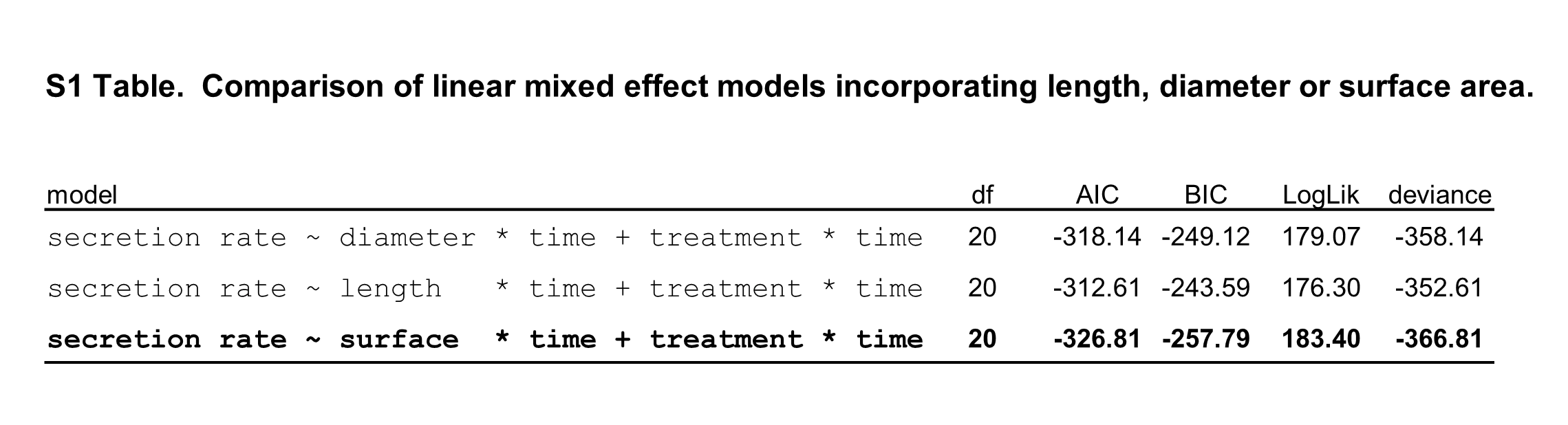

Supplement: S1 Table — Based on the lowest AIC parameter, the surface area was the best explanatory variable for the secretion rate. The row in bold indicates the model with the lowest AIC. Only the fixed effects are shown. (TIF) [file pone.0223569.s007.tif]

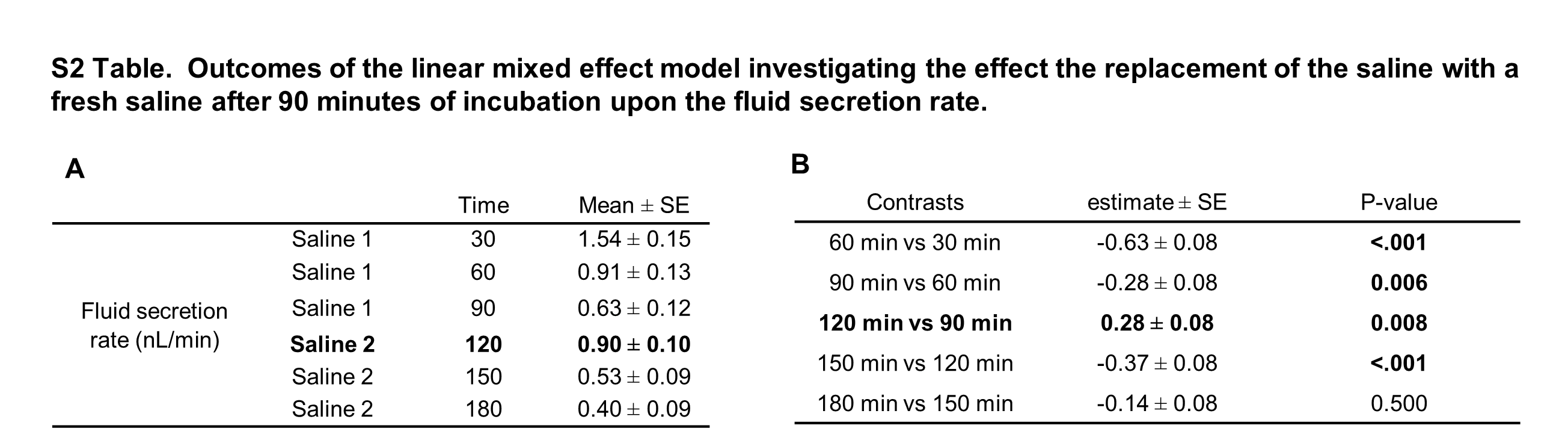

Supplement: S2 Table — The model applied was (Secretion rate ~ surface + time + (1| locust) + (1+time|tubule)). The rows in bold indicate the first observation after the saline has been replaced. (A) Summary of the mean values of fluid secretion rate at each time point. (B) Pairwise comparisons between subsequent times of incubation. (TIF) [file pone.0223569.s008.tif]

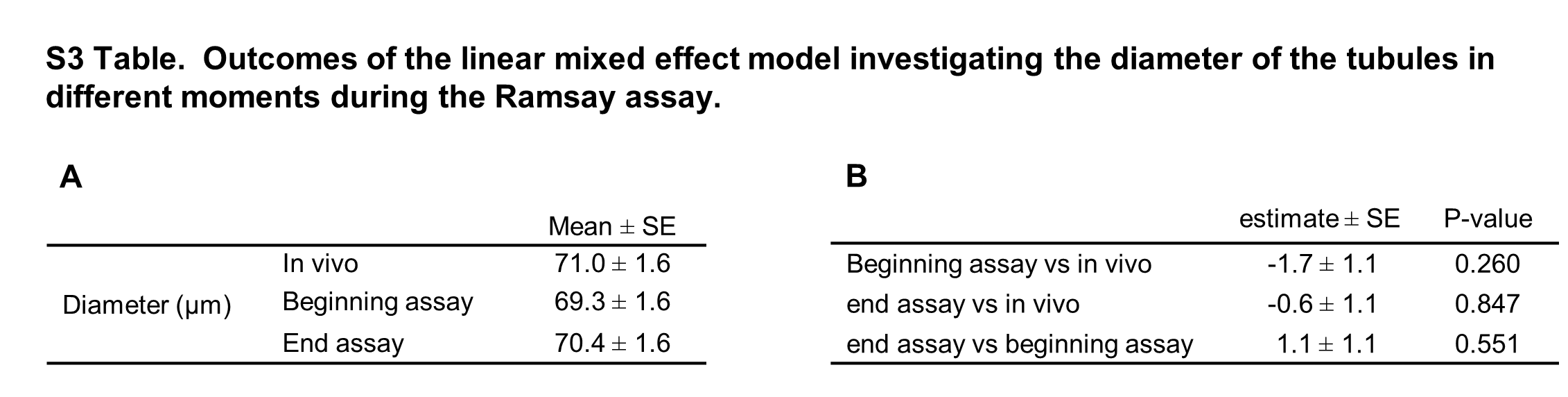

Supplement: S3 Table — The model applied was (diameter ~ assay time + (1| locust) + (1+time|tubule)). (A) Summary of the mean diameter of Malpighian tubules in vivo, at the beginning of the assay and at the end of the assay after 180 minutes of incubation. (B) Pairwise comparisons between different moments of the assay. (TIF) [file pone.0223569.s009.tif]
